# Assessing the *in vivo* ameliorative effects of *Lactobacillus acidophilus* KLDS1.0901 for induced non-alcoholic fatty liver disease treatment

**DOI:** 10.3389/fnut.2023.1147423

**Published:** 2023-03-20

**Authors:** Yanbo Wang, Zengbo Wang, Yang Wan, Furong Jin, Xiaodan Shi, Zhishuang Xing, Bo Tian, Bailiang Li

**Affiliations:** ^1^College of Food, Northeast Agricultural University, Harbin, China; ^2^Key Laboratory of Dairy Science, Ministry of Education, Northeast Agricultural University, Harbin, China

**Keywords:** *Lactobacillus acidophilus*, high-fat diet, non-alcoholic fatty liver disease, lipid accumulation, gut microbiota

## Abstract

Reputed as a significant metabolic disorder, non-alcoholic fatty liver disease (NAFLD) is characterized by high-fat deposits in the liver and causes substantial economic challenges to any country's workforce. Previous studies have indicated that some lactic acid bacteria may effectively prevent or treat NAFLD. Overall, *L. acidophilus* KLDS1.0901 protected against HFD-induced NAFLD by improving liver characteristics and modulating microbiota composition, and thus could be a candidate for improving NAFLD. This study aimed to assess the protective effects of *L. acidophilus* KLDS1.0901 on a high-fat diet(HFD)-induced NAFLD. First, hepatic lipid profile and histological alterations were determined to study whether *L. acidophilus* KLDS1.0901 could ameliorate NAFLD. Then, the intestinal permeability and gut barrier were explored. Finally, gut microbiota was analyzed to elucidate the mechanism from the insights of the gut–liver axis. The results showed that *Lactobacillus* KLDS1.0901 administration significantly decreased body weight, Lee's index body, fat rate, and liver index. *L. acidophilus* KLDS1.0901 administration significantly improved lipid profiles by decreasing the hepatic levels of total cholesterol (TC), triglyceride (TG), and low-density lipoprotein cholesterol (LDL-C) and by increasing the high-density lipoprotein cholesterol (HDL-C) levels. A conspicuous decrease of alanine aminotransferase (ALT) and aspartate aminotransferase (AST) in serum was observed after *L. acidophilus* KLDS1.0901 administration. Meanwhile, the H&E and Oil Red O-stained staining showed that *L. acidophilus* KLDS1.0901 significantly reduced liver lipid accumulation of HFD-fed mice by decreasing the NAS score and lipid area per total area. Our results showed that *L. acidophilus* KLDS1.0901 administration decreased the interleukin-6 (IL-6), interleukin-1β (IL-1β), and tumor necrosis factor-alpha (TNF-α) concentrations accompanied by the increase of interleukin-10 (IL-10). *L. acidophilus* KLDS1.0901 administration could improve the intestinal barrier function by upregulating the mRNA levels of occludin, claudin-1, ZO-1, and Muc-2, which were coupled to the decreases of the concentration of LPS and D-lactic acid. Notably, *L. acidophilus* KLDS1.0901 administration modulated the gut microbiota to a near-normal pattern. Hence, our results suggested that *L. acidophilus* KLDS1.0901 can be used as a candidate to ameliorate NAFLD.

## Introduction

Soaring obesity rates have been closely linked to non-communicable metabolic diseases like chronic liver and cardiovascular diseases as approximately 90% of patients are diagnosed with severe obesity and non-alcoholic fatty liver disease (NAFLD) (1, 2). NAFLD is a highly prevalent liver disease characterized by non-frequent alcohol consumption (3, 4) and could progress to serious conditions such as liver fibrosis, cancer, and cirrhosis (52). Because the pathogenesis of NAFLD is still unclear, studies are ongoing to develop an effective treatment protocol for NAFLD (3). Natural products incorporated into diets (ginkgolide C, polyphenols, yeast-fermented wall-broken bee pollen, among others) could be effective against NAFLD (5–7). Furthermore, next-generation sequencing techniques have revealed a possible correlation between NAFLD pathogenesis and changes in the intestinal microbiome (8), thus offering a potential strategy for diet-induced NAFLD (9).

As a widely used probiotic in foods, *Lactobacillus acidophilus* exerts several benefits after proliferating in the gastrointestinal tract, including ameliorating type 2 diabetes, enteric infections, allergic dermatitis, and renal failure and hepatic failure, various forms of inflammatory bowel disease, lactose intolerance, and possible anticancer activity. Furthermore, other studies have reported its potential to control serum cholesterol concentrations, reduce tumor development risks, and ensure better digestion to boost host immunity (10–12). Researchers have been paying more attention to *L. acidophilus* to improve abnormal glucose and lipid metabolism, especially NAFLD. Andreasen et al. reported that *L. acidophilus* NCFM could improve insulin sensitivity and the systemic inflammatory response in human subjects (52). It has been found that *L. acidophilus* LA5 could improve the saturated fat-induced obesity mouse model through the enhanced intestinal *Akkermansia muciniphila* (5). *L. acidophilus* NX2-6 showed the potential against oleic acid-induced steatosis, mitochondrial dysfunction, endoplasmic reticulum stress, and inflammatory responses (6). *L. acidophilus* SNZ 86 could alleviate Western diet-induced non-alcoholic fatty liver disease in rats *via* modulation of autophagy through the AMPK/SIRT-1 pathway (7). An Egyptian study by Abdel Monem with Zigazag University randomized patients with NASH to probiotic Acidophilus capsule (2 billion *Lactobacillus acidophilus*) or placebo for 17 months and measured improved AST and ALT in treated patients (8). *L. acidophilus* showed a significant reduction in the liver/body weight ratio and a significant improvement in steatosis compared to the patients with NAFLD (9). Yogurt fermented with *L. acidophilus* improves body mass index and fasting insulin levels without affecting serum leptin and adiponectin levels in NAFLD (10).

*Lactobacillus acidophilus* KLDS1.0901 was isolated from traditional fermented dairy products in Sinkiang Province, China, and preserved in our laboratory. Our previous studies showed *L. acidophilus* KLDS1.0901 with antioxidative activity had good tolerance to acid and bile salt and strong adhesion ability (11). Furthermore, *L. acidophilus* KLDS1.0901 could alleviate type 2 diabetes by regulating hepatic glucose, lipid metabolism, and gut microbiota in mice (12). *L. acidophilus* KLDS1.0901 also could prevent chronic alcoholic liver injury in mice by protecting the intestinal barrier and regulating gut microbiota and liver-related pathways (13). Thus, we hypothesized that *L. acidophilus* KLDS1.0901 would possess the ability to alleviate NAFLD. The aim of this study was to assess the protective effects of *L. acidophilus* KLDS1.0901 on HFD-induced NAFLD. First, hepatic lipid profile and histological alterations were determined to study whether *L. acidophilus* KLDS1.0901 could ameliorate NAFLD. However, the intestinal permeability and gut barrier were explored. Finally, gut microbiota was analyzed to elucidate the mechanism from the insights of the gut–liver axis.

## Materials and methods

### Bacterial strain and culture

Traditional fermented dairy products from Xinjiang Province, China were used to obtain *Lactobacillus acidophilus* KLDS 1.0901 and stored in 20% (v/v) glycerol at −20°C. The bacteria was incubated for 18 h in de Man Rogosa and Sharpe (MRS) broth (2% v/v) at 37°C and subcultured two times. Bacterial cultures were then centrifuged (6,000 rpm for 10 min at 4°C), washed three times with PBS solution, and the supernatant discarded. Cells were then resuspended in PBS at 5 × 10^9^ CFU/mL.

### Animals and experiment design

Following the method of Nguyen et al. (13), 6-week-old male C57BL/6J mice (*n* = 24) were obtained from the Vital River Laboratory Animal Technology Company (Beijing, China). Study animals were housed in a sterile animal room at 22 ±0.5°C, 55±5% humidity with 12 h light/12 h dark cycles. They had *ad libitum* access to chow and water *ad libitum* throughout the study. Following a 7-day acclimatization phase, mice were randomly divided into three groups (*n* = 8 mice per group). Control group (NC) mice were fed a D12450B diet, while others were fed a D12492 high-fat diet for 8 weeks. Feed formulas are reported in Supplementary Table S1. Both groups were gavaged with 0.2 mL of sterile PBS solution, and for the *L. acidophilus* KLDS1.0901 group (KLDS1.0901), the mice were administered with 0.2 mL of the *L. acidophilus* KLDS1.0901 (10^9^ CFU/d). Study animals were humanely sacrificed after 12 h of fasting and blood samples were obtained. Colon and liver samples as well as colon and cecum content were collected and stored at−80°C for further analysis. The Northeast Agricultural University Guide for the Care and Use of Laboratory Animals was followed to ensure strict ethical animal procedures. In addition, our study was approved by the Northeast Agricultural University Animal Ethics Committee.

### Histopathological analysis of liver

In reference to the previous method with a slight modification (14), we placed all liver tissues in paraffin, in thin slices of 5 μm thick, and stained with hematoxylin and eosin (H&E) and oil O red after deparaffinization. The sections were observed under a light microscope (Nikon E100, 200 × magnification) for lesions and other histological features. The lipid droplet area (percentage of total area) of each group was analyzed using the software Image J. NAFLD activity integral was calculated based on Supplementary Table S2.

#### Determination of TC, TG, LDL-C, and HDL-C in liver

The liver tissues were homogenized in aseptic PBS (1:9, w/v), succeeded by centrifugation at 10,000 rpm for 10 min at 4°C. The concentration of TC, TG, HDL-C, and LDL-C was detected using mouse ELISA kits (Conodi creatures, Fujian, China) based on the instructions of the manufacturer.

#### Determination of ALT and AST in Serum

Each mice group's serum AST and ALT levels were obtained using mouse kits (Conodi Creatures, Fujian, China) by following the manufacturer's directives.

#### ELISA measurement of inflammatory cytokines

Aseptic PBS (1:9, w/v) was used to homogenize mice liver tissues, followed by centrifugation (10,000 rpm, 10 min, 4°C). Based on the manufacturer's directives, we identified the concentration of TNF-α, IL-6, IL-10, and IL-1β with mouse ELISA kits (Conodi creatures, Fujian, China).

### Measurement of the intestinal permeability

Following the manufacturer's instructions, the Enzyme-Linked Immunosorbent Assay (ELISA) kits (Conodi creatures, Fujian, China) were used to evaluate the concentrations of D-lactic acid (D-LA) and lipopolysaccharide (LPS) in mice serum samples. The serum samples were acquired before the mice were sacrificed.

### Real-time quantitative polymerase chain reaction (RT-qPCR) analysis

Real-time quantitative polymerase chain reaction was used to determine relative mRNA expression levels of tight junction protein ZO-1, claudin-1, occludin, and Muc-2. Colon tissue RNA levels were obtained and quantified using the Total RNA kit (Vazyme, Nanjing, China), and 2000C Ultra-micro UV spectrophotometer (Thermo Fisher Scientific Inc., USA), respectively. We used the Transcriptor First Strand cDNA Synthesis kit (Promega, Madison, USA) to synthesize cDNA for this study. The GoTaq°R SYBR-Green qPCR Master Mix (Promega, Madison, USA) was used to perform RT-qPCR corrections. The relative mRNA expressions of specific genes were calculated by the 2^−ΔΔCT^ method. GAPDH genes are used as internal reference genes. Supplementary Table S3 displays the specific gene primers as designed by Sangon Biotech Co., Ltd (Shanghai, China).

### DNA extraction and 16S rRNA gene sequencing

Fecal samples served as the substrate for the total bacterial genomic DNA using the Fast DNA SPIN extraction kits (MP Biomedicals, Santa Ana, CA, USA). DNA molecular size and quantification were performed using a 0.8% agarose gel electrophoresis and the NanoDrop NC-2000 spectrophotometer, respectively. The V3–V4 region of bacterial 16S rRNA genes was then amplified using PCR with the forward primer 338F (5'-ACTCCTACGGGAGGCAGCA-3') and the reverse primer 806R (5'-GGACTACHVGGGTWTCTAAT-3'). PCR amplicons were purified with Agencourt AMPure Beads (Beckman Coulter, Indianapolis, IN) and quantified using the PicoGreen dsDNA Assay Kit (Invitrogen, Carlsbad, CA, USA). Finally, the MiSeq Reagent kit v3 (Shanghai Personal Biotechnology Co., Ltd, Shanghai, China) was used to carry out the sequencing on the Illumina MiSeq platform.

### Statistical analysis

All data were analyzed using SPSS 22.0 software (SPSS Inc., Chicago, IL, USA). Statistical analysis of Duncan's multiple range tests after one-way analysis of variance (ANOVA). For all analyses, at *p* < 0.05, the differences were considered significant.

## Results

### Effect of *L. acidophilus* KLDS1.0901 administration on body weight, body fat rate, Lee's index, and liver index of HFD-fed mice

**Figure 1:**
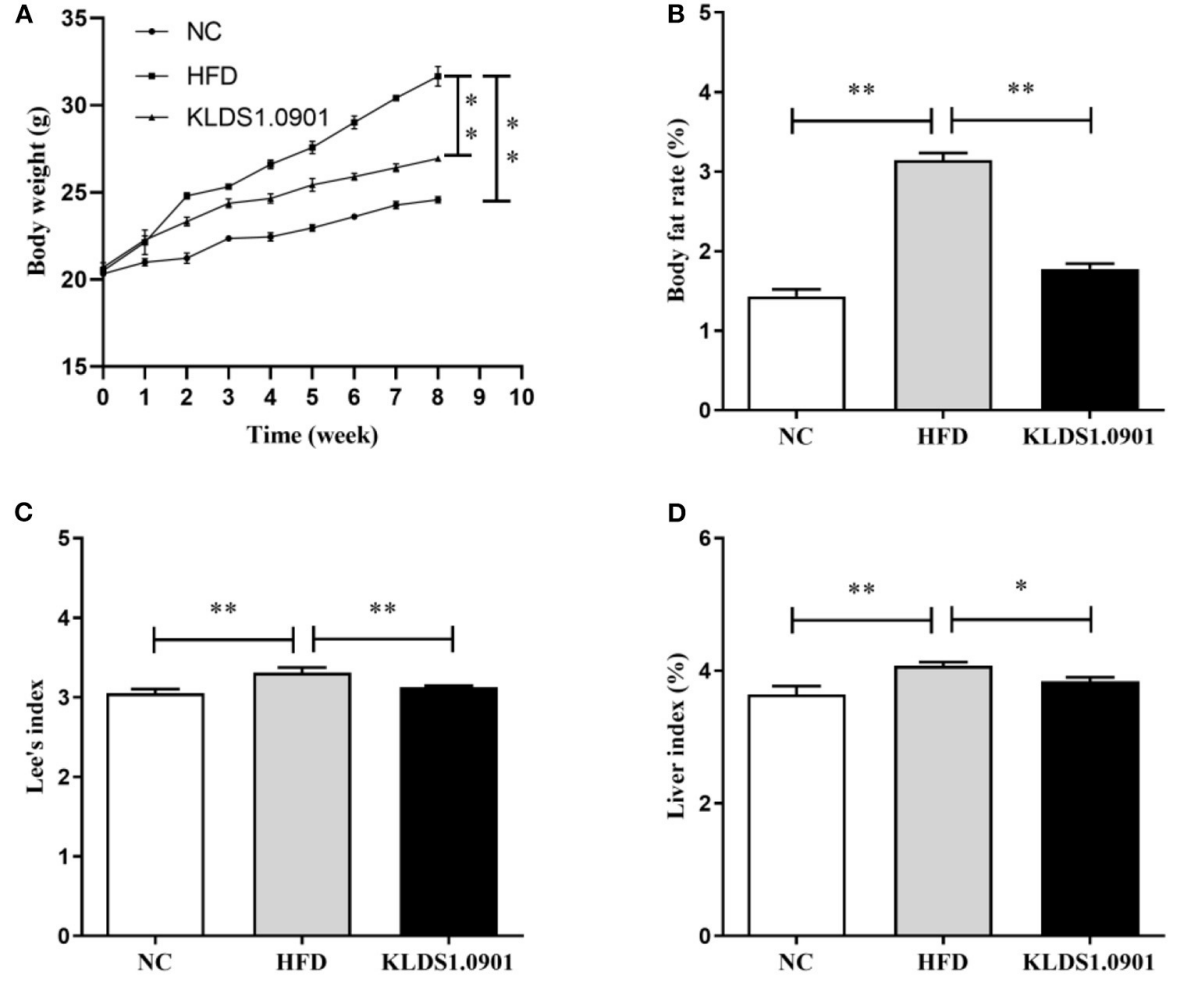
Effect of *L. acidophilus* KLDS1.0901 administration on body weight **(A)**, body fat rate **(B)**, Lee's index **(C)**, and liver index **(D)** of HFD-fed mice. NC, normal control group; HFD, high-fat diet group; and KLDS1.0901, *L. acidophilus* KLDS1.0901 group. *p < 0.05 and **p < 0.01 indicated that there was a significant difference when compared with the HFD group.

High-fat diet significantly increased (*p* < 0.01) the final body weight, whereas the *L. acidophilus* KLDS1.0901 administration reduced (*p* < 0.01) it when compared with the HFD group (Figure 1A). The body fat rate of the mice in the HFD group was sharply elevated when compared to that of the NC group (*p* < 0.01), however, *L. acidophilus* KLDS1.0901 administration inhibited the increase (*p* < 0.01) (Figure 1B). Lee's index in the HFD group was higher (*p* < 0.01) than that in the control group, but Lee's index was remarkably decreased (*p* < 0.01) after *L. acidophilus* KLDS1.0901 administration (Figure 1C). In the analysis of the liver index, compared with the NC group, mice in the HFD group showed a marked increase (*p* < 0.01), which was reversed (*p* < 0.05) by the *L. acidophilus* KLDS1.0901 administration (Figure 1D).

### Effect of *L. acidophilus* KLDS1.0901 administration on lipid accumulation and liver function in HFD-fed mice

**Figure 2:**
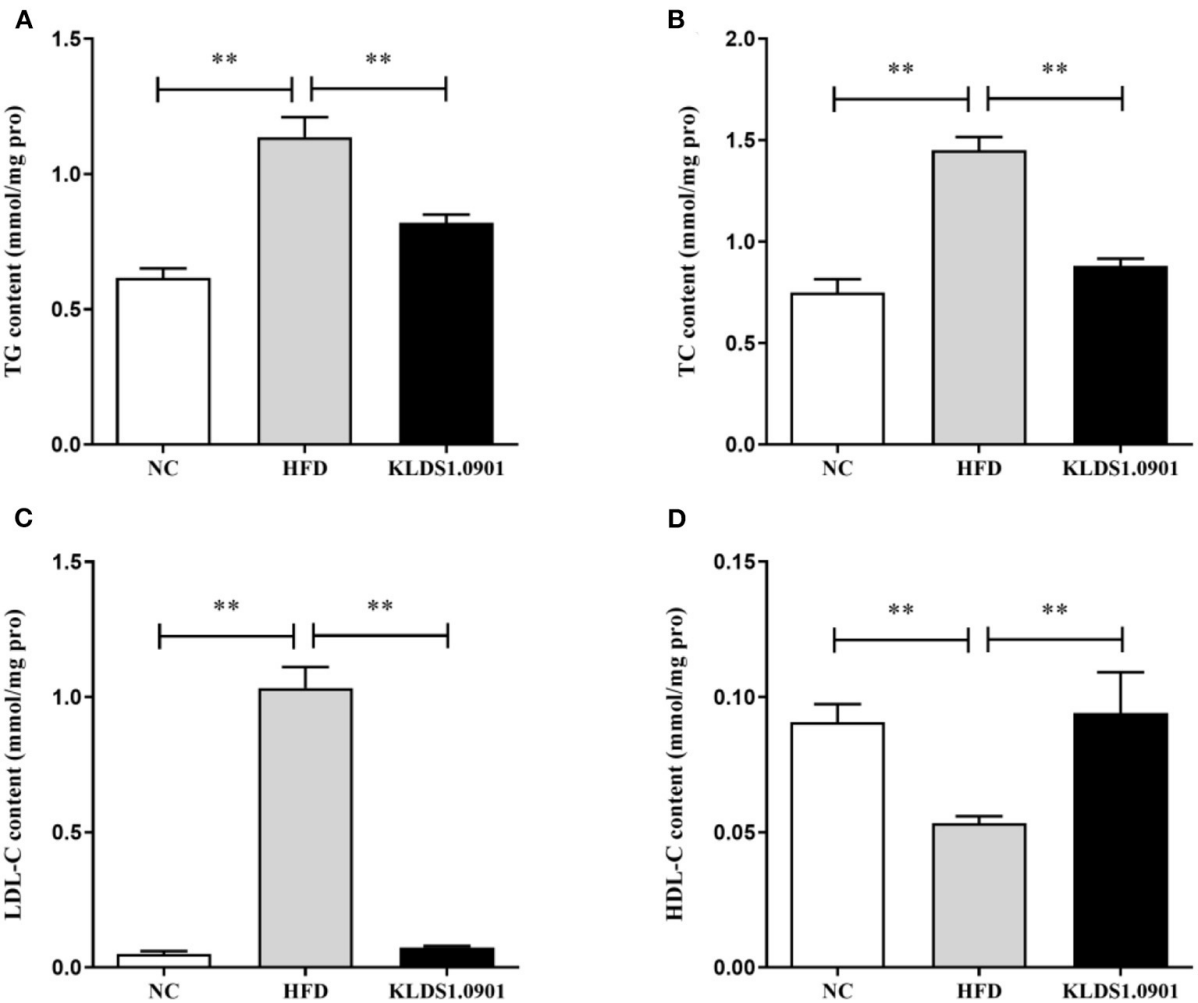
Effects of *L. acidophilus* KLDS1.0901 administration on lipid accumulation in HFD-fed mice. NC, normal control group; HFD, high-fat diet group; and KLDS1.0901, *L. acidophilus* KLDS1.0901 group. **(A)** Hepatic triglyceride (TG) level; **(B)** hepatic total cholesterol (TC) level; **(C)** hepatic low-density lipoprotein cholesterol (HDL-C) level; and **(D)** hepatic high-density lipoprotein cholesterol (LDL-C) level. Values are expressed as mean ± SD (*n* = 8). **p < 0.01 indicated that there was a significant difference when compared with the HFD group.

**Figure 3:**
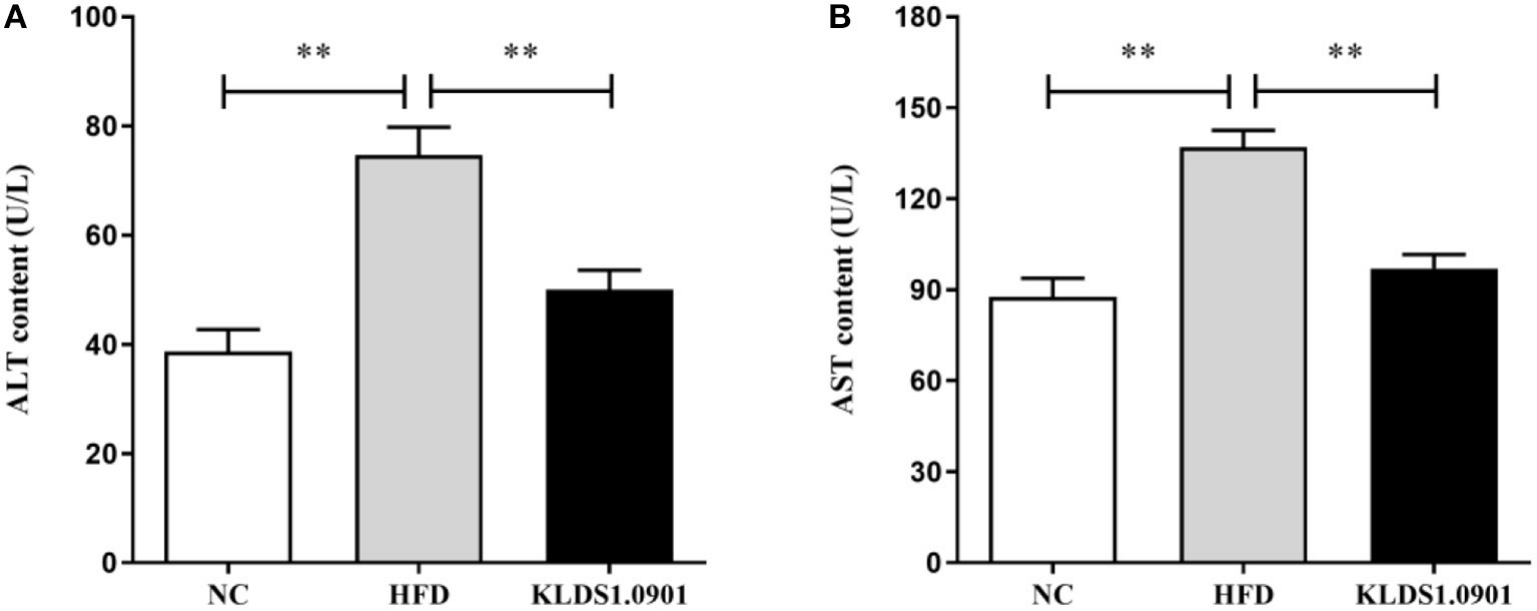
Effects of *L. acidophilus* KLDS1.0901 administration on liver function. NC, normal control group; HFD, high-fat diet group; and KLDS1.0901, *L. acidophilus* KLDS1.0901 group. **(A)** Serum alanine aminotransferase (ALT) level; and **(B)** serum aspartate aminotransferase (AST) level. Values are expressed as mean ± SD(*n* = 8). **p < 0.01 indicated that there was a significant difference when compared with the HFD group.

To analyze hepatic lipid accumulation, the levels of TG, TC, LDL-C, and HDL-C in the liver of mice were determined by ELISA. As shown in Figures 2A–D. The lipid profiles including TG, TC, and LDL-C were pronouncedly (*p* < 0.01) enhanced in the HFD group when compared with the control group. However, *L. acidophilus* KLDS1.0901 administration suppressed the increases in these lipid parameters (*p* < 0.01). On the contrary, the HDL-C level was significantly lowered (*p* < 0.01) in the HFD group when compared with that in the NC group, *L. acidophilus* KLDS1.0901 administration reversed this trend (*p* < 0.01). As shown in Figures 3A, B. In order to study liver function, the serum ALT and AST levels of mice were examined by ELISA. A significant increase (*p* < 0.01) of ALT and AST in serum was observed in the HFD group. Notably, *L. acidophilus* KLDS1.0901 administration conspicuously inhibited this effect (*p* < 0.01).

### Effects of *L. acidophilus* KLDS1.0901 administration on histological alterations of liver

**Figure 4:**
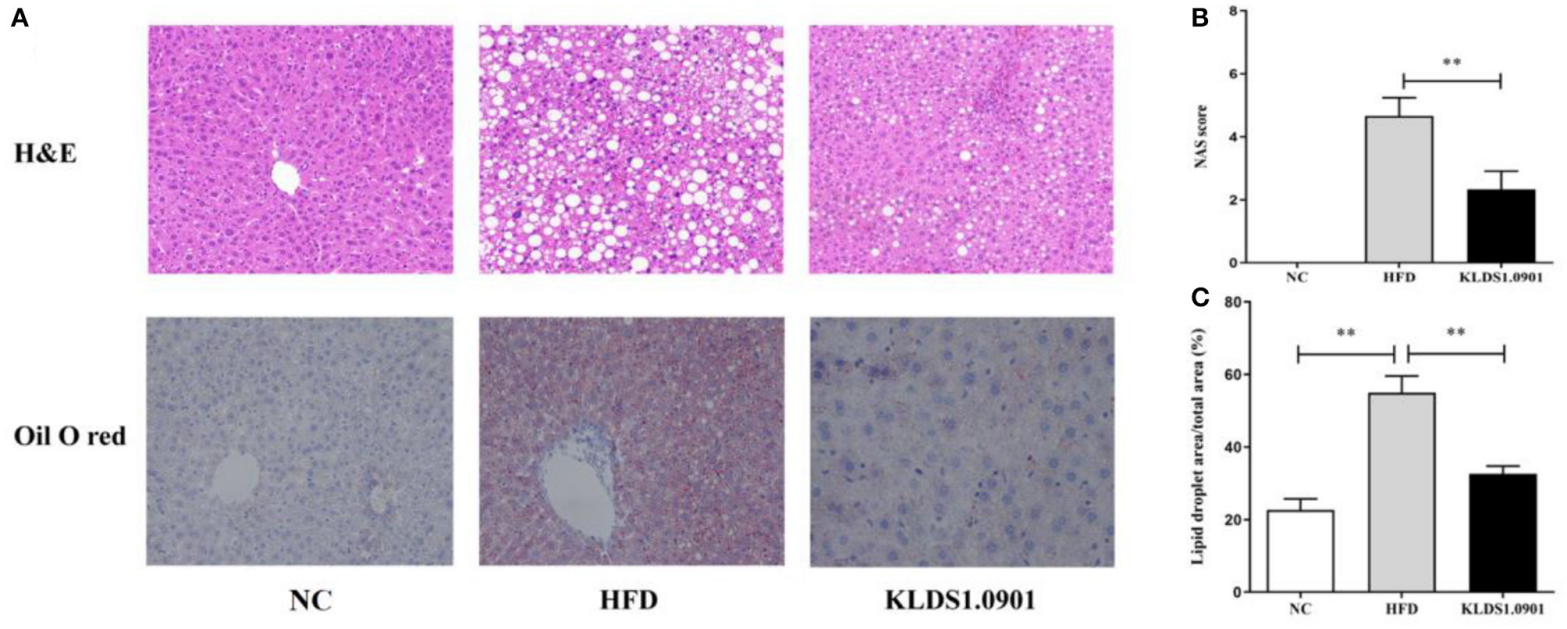
Effects of *L. acidophilus* KLDS1.0901 administration on histological alterations of the liver. NC, normal control group; HFD, high-fat diet group; and KLDS1.0901, *L. acidophilus* KLDS1.0901 group. **(A)** The representative histological changes of liver sections; **(B)** NAFLD Activity Score (NAS); and **(C)** quantification of Oil Red O-stained hepatic lipid droplets. Values are expressed as mean ± SD (*n* = 8). **p < 0.01 indicated that there was a significant difference when compared with the HFD group.

In the NC group, there was no steatosis, and the tissue structure was clear and complete. The H&E staining showed that HFD blurred the boundary and induced the regular round fat hole. In particular, HFD detrimentally caused substantial fat accumulation in the liver during 8 weeks of feeding, whereas *L. acidophilus* KLDS1.0901 administration effectively restored the trend caused by HFD (Figure 4A). As shown in Figure 4B, in view of the NAFLD activity score (NAS), the HFD-induced increase was significantly (*p* < 0.01) reduced by *L. acidophilus* KLDS1.0901 administration. Quantification of lipid area per total area also suggested that *L. acidophilus* KLDS1.0901 administration significantly reduced (*p* < 0.01) liver lipid accumulation of HFD-fed mice (Figure 4C).

### Effect of *L. acidophilus* KLDS1.0901 administration on hepatic inflammation

**Figure 5:**
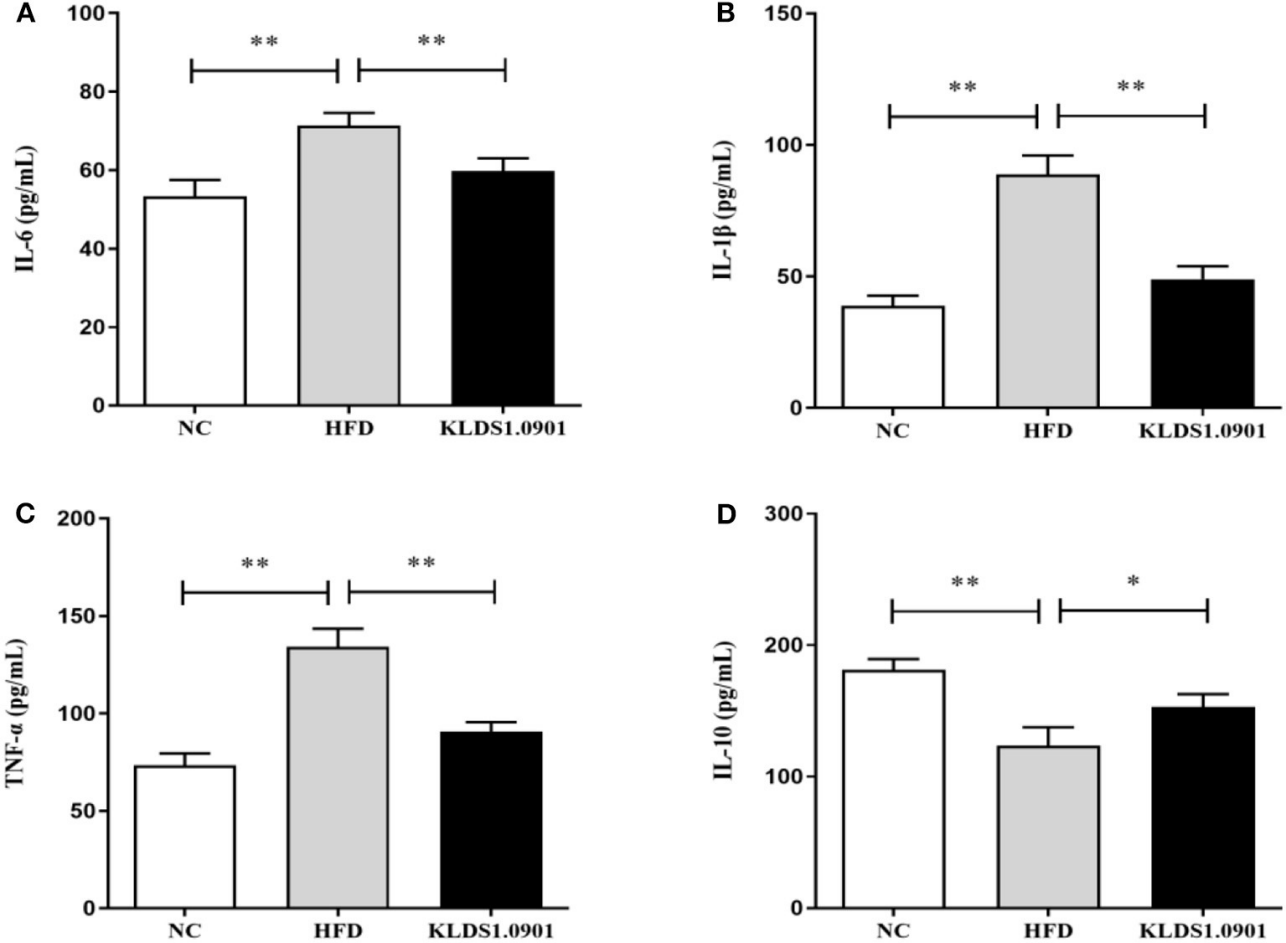
Effects of *L. acidophilus* KLDS1.0901 administration on hepatic inflammation. NC, normal control group; HFD, high-fat diet group; and KLDS1.0901, *L. acidophilus* KLDS1.0901 group. **(A)** IL-6; **(B)** IL-1β; **(C)** TNF-α; and **(D)** IL-10. Values are expressed as mean ± SD (*n* = 8). *p < 0.05 and **p < 0.01 indicated that there was a significant difference when compared with the HFD group.

As shown in Figure 5, our cytokine analyses showed that the concentrations of IL-6, IL-1β, and TNF-α increased considerably in the HFD mice compared to the NC group (*p* < 0.01); however, *L. acidophilus* KLDS1.0901 administration significantly decreased them (*p* < 0.01). While the concentration of IL-10 was significantly decreased (*p* < 0.01), *L. acidophilus* KLDS1.0901 administration significantly increased it (*p* < 0.05).

### Effect of *L. acidophilus* KLDS1.0901 administration on the intestinal permeability and gut barrier

**Figure 6:**
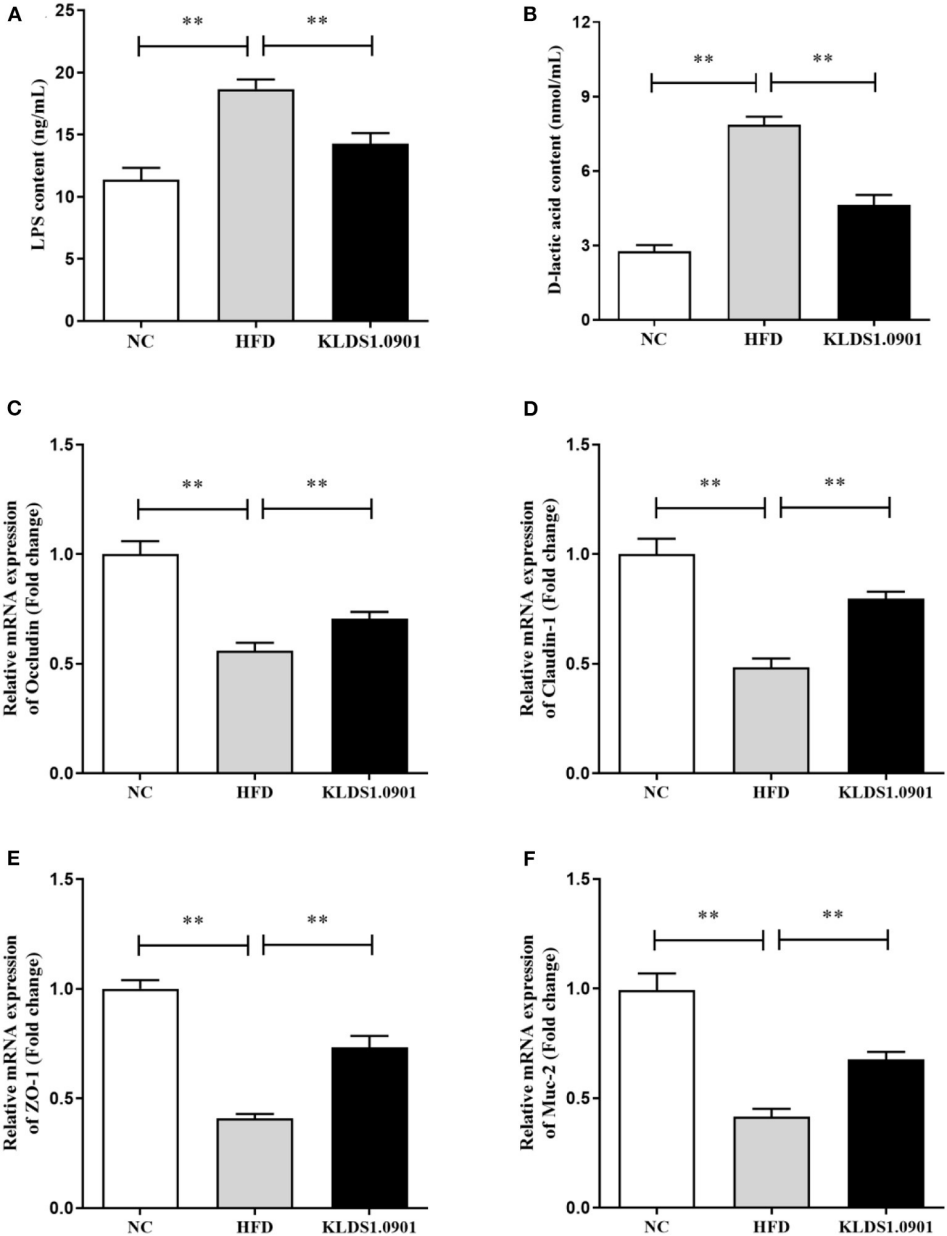
Effects of *L. acidophilus* KLDS1.0901 administration on the intestinal permeability and gut barrier. NC, normal control group; HFD, high-fat diet group; and KLDS1.0901, *L. acidophilus* KLDS1.0901 group. **(A)** Serum LPS concentration; **(B)** serum D-lactic acid concentration; **(C)** occludin; **(D)** claudin-1; **(E)** ZO-1; and **(F)** Muc-2. Values are expressed as mean ± SD (*n* = 8). **p < 0.01 indicated that there was a significant difference when compared with the HFD group.

Lipopolysaccharides and D-lactic acid are standard indicators of intestinal barrier damage, the concentration of LPS and D-lactic acid was examined by ELISA. As shown in Figures 6A, B, the concentration of LPS and D-lactic acid increased substantially (*p* < 0.01) in the HFD group, resulting in the anticipated mucosal damage, but *L. acidophilus* KLDS1.0901 administration drastically reduced (*p* < 0.01) the concentration of LPS and D-lactic acid, thus improving intestinal permeability. To study the effect of *L. acidophilus* KLDS1.0901 administration on the gut barrier, we determined the mRNA levels of occludin, claudin-1, ZO-1, and Muc-2. The results showed that the mRNA levels of these four genes were strikingly downregulated (*p* < 0.01) in the HFD group, but *L. acidophilus* KLDS1.0901 administration significantly upregulated (*p* < 0.01) the mRNA levels of these four genes (Figures 6C–F).

### Effect of *L. acidophilus* KLDS1.0901 administration on gut microbiota

**Figure 7:**
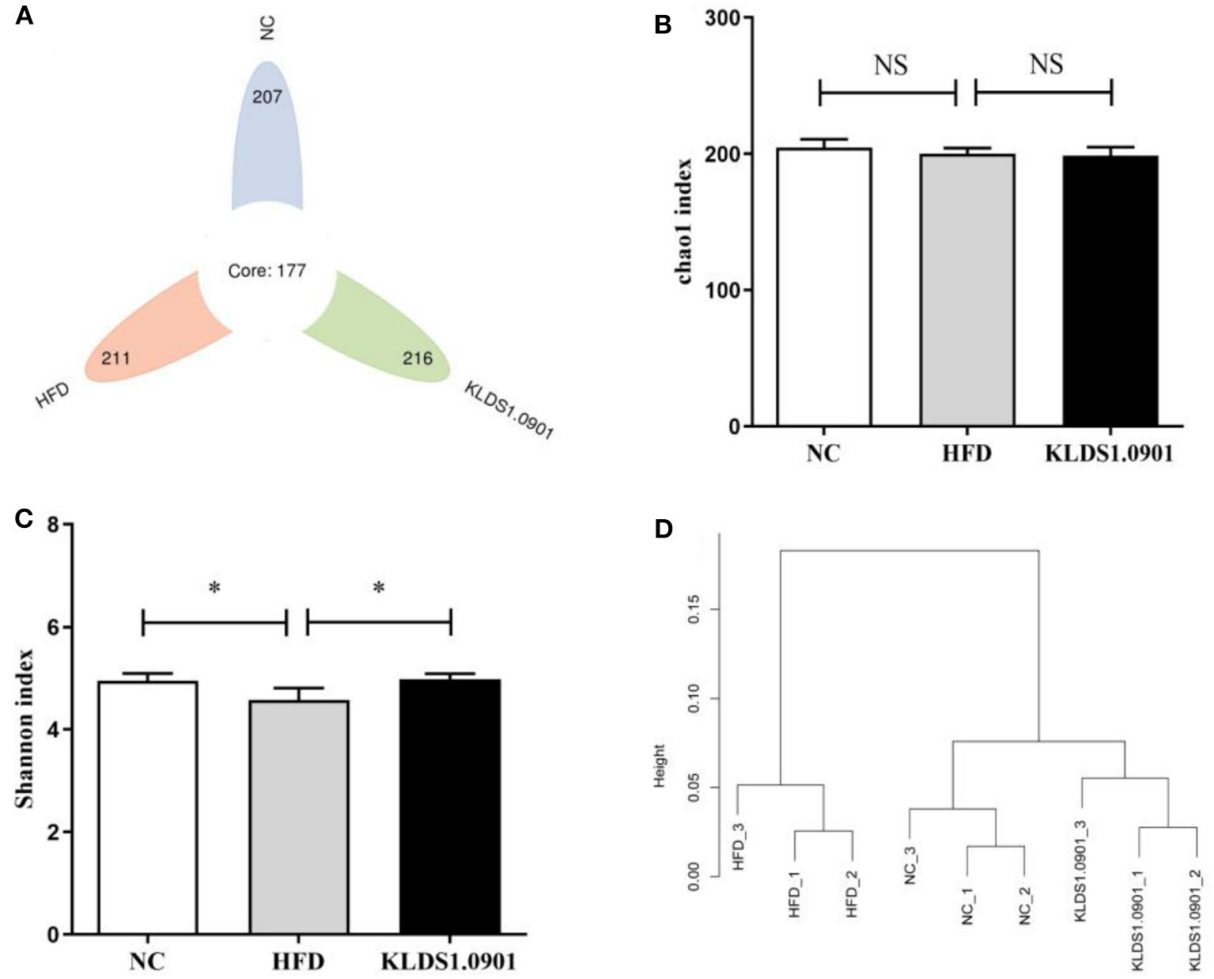
*L. acidophilus* KLDS1.0901 administration regulates **(A)** the number of OTUs, **(B)** Chao 1 index, **(C)** Shannon index, and **(D)** the hierarchical clustering tree of weighted UniFrac distances. NC, normal control group; HFD, high-fat diet group; and KLDS1.0901, *L. acidophilus* KLDS1.0901 group. Values are expressed as mean ± SD (*n* = 8). *p < 0.05 indicated that there was a significant difference when compared with the HFD group. NS indicated that there was no significant difference when compared with the HFD group.

We sequenced the 16S rDNA V3–V4 variable region to analyze the cecal gut microbiota. This enabled us to gain insights as to whether *L. acidophilus* KLDS1.0901 administration modulated the bacterial communities of NAFLD-induced mice. The results showed that the OTU determined the gut microbiota diversity of each study group, with the common abundance shown with a Venn diagram (177 OTU in all groups). Our results showed 207, 211, and 216 different microorganisms in the NC, HFD, and MC groups, respectively (Figure 7A). The α-diversity reflecting the microbial community diversity was assessed by the Chao 1 and Shannon indexes (Figures 7B, C). There were no significant changes in Chao 1 index among the three groups. However, the Shannon index in the HFD mice was lower than that in the NC group (*p* < 0.05), which was significantly elevated by *L. acidophilus* KLDS1.0901 administration (*p* < 0.05). The hierarchical clustering tree results of the current study grouped the NC and KLDS 1.0901 cohorts together and then clustered with the HFD communities (Figure 7D).

**Figure 8:**
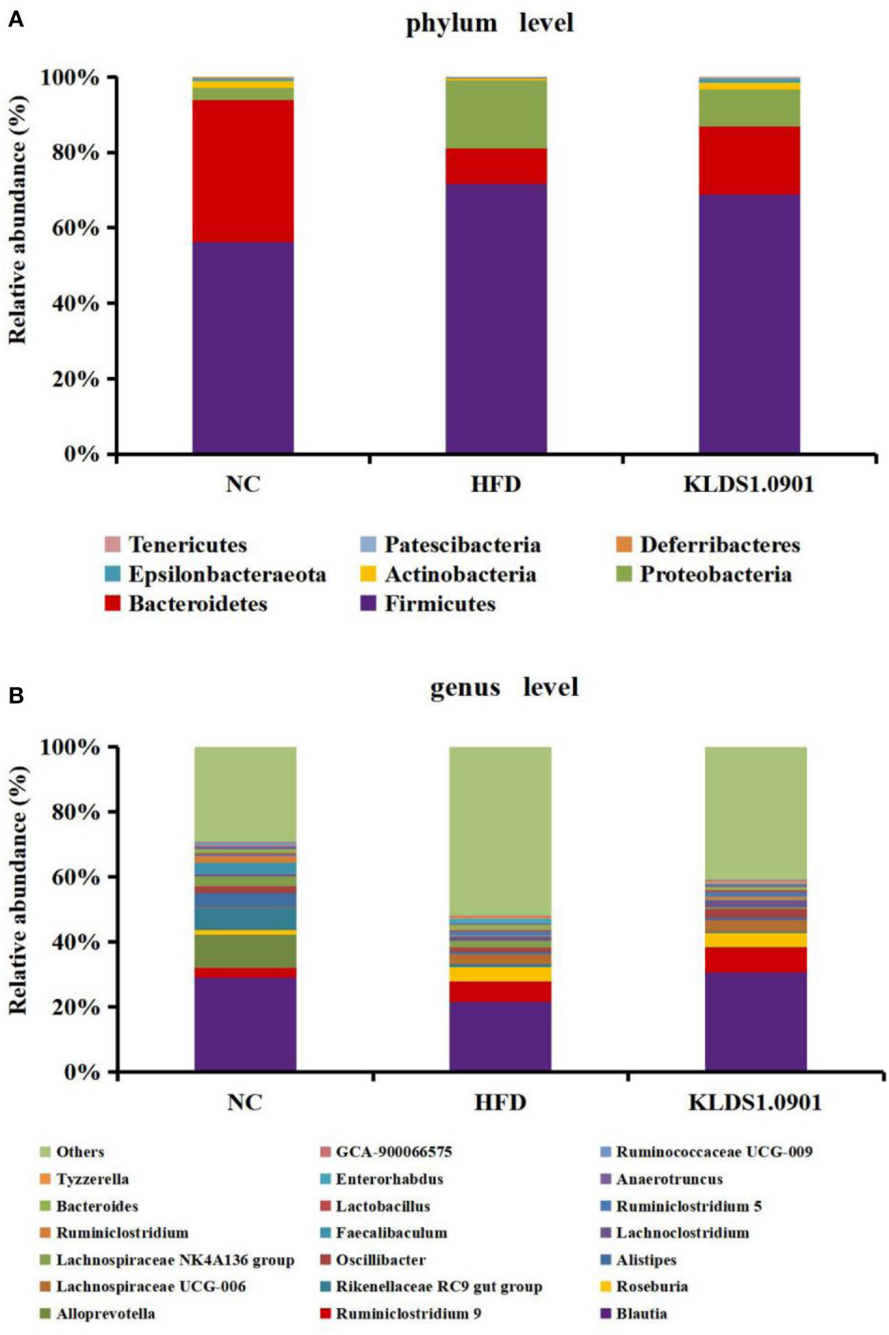
Changes of the gut microbiota at the phylum **(A)** and genus **(B)** level after *L. acidophilus* KLDS1.0901 administration. NC, normal control group; HFD, high-fat diet group; and KLDS1.0901, *L. acidophilus* KLDS1.0901 group.

Our phylum-level results indicated that Firmicutes and Bacteriodetes make up at least 80% of all groups (Figure 8A). The HFD group had a high abundance of Firmicutes and depleted levels of Bacteriodetes. This group also had a significantly high ratio of Firmicutes to Bacteriodetes. We also observed that *L. acidophilus* KLDS1.0901 administration reversed this trend by lowering Firmicutes levels, increasing Bacteroidetes abundance, and reducing the HFD-induced Firmicutes-to-Bacteroidetes ratio.

The genus-level gut microbiota results for the different groups are shown in Figure 8B. We observed that in the HFD group, the relative abundances of *Roseburia, Lachnospiraceae UCG-006, Bacteroides*, and *Enterorhabdus* were much higher than that in the NC group, which were suppressed by *L. acidophilus* KLDS1.0901 administration. However, the HFD group had low levels of *Blautia, Alistipes, Oscillibacter, Faecalibaculum, Ruminiclostridium, Lactobacillus*, and *Ruminococcaceae UCG-009*, which were reversed by *L. acidophilus* KLDS1.0901 administration. Our observations indicated that *L. acidophilus* KLDS1.0901 administration modulated the composition of the intestinal microorganisms of HFD-fed mice.

## Discussion

The prevalence of NAFLD has been linked to the onset and exacerbation of other metabolic disorders like type 2 diabetes (T2D) and obesity, thus posing a significant public health concern. It has been posited previously that the progression of NAFLD could be driven by lipid metabolism disorders (2). Accumulating evidence suggests that some lactic acid bacteria have the potential to relieve NAFLD (13, 15). Our previous studies showed *L. acidophilus* KLDS1.0901 with antioxidative activity had good tolerance to acid and bile salt and strong adhesion ability (11). Furthermore, *L. acidophilus* KLDS1.0901 could alleviate type 2 diabetes by regulating hepatic glucose, lipid metabolism, and gut microbiota in mice (12). *L. acidophilus* KLDS1.0901 also could prevent chronic alcoholic liver injury in mice by protecting the intestinal barrier and regulating gut microbiota and liver-related pathways (13). However, the underlying mechanisms were unclear. Thus, in this study, the possible mechanisms by which the same lactobacilli strains could prevent obesity were mined and increased the final body weight, body fat rate, Lee's index, and liver index.

In this study, HFD significantly increased the final body weight, body fat rate, Lee's index, and liver index, which was reversed by the *L. acidophilus* KLDS1.0901 administration, which was in line with the results of Naudin et al. (16), expressing *L. acidophilus* KLDS1.0901 had the protective effects of HFD-induced NAFLD. HFD (manifested in hyperlipidemia) is known to trigger NAFLD by elevating TC, TG, and LDL-C levels, with a corresponding drop in host HDL-C levels (17). Interestingly, some prior studies have confirmed that reducing TC, TG, and LDL-C levels can alleviate NAFLD (18, 19, 51). In this study, the HFD significantly elevated the hepatic levels of TC, TG, and LDL-C and lowered the HDL-C levels, resulting in lipid metabolism disorder. However, *L. acidophilus* KLDS1.0901 administration reversed this trend and agrees with the effects of *L. plantarum* NA136 and *L. johnsonii* BS15 as reported earlier (20, 21). Meanwhile, the H&E and Oil Red O-stained staining showed that HFD detrimentally caused substantial fat accumulation in the liver during 8 weeks of feeding and increased the NAS score and lipid area per total area. These results indicated that *L. acidophilus* KLDS1.0901 administration could have protective effects from hepatic steatosis due to lowered lipid content in NAFLD mice.

Furthermore, elevated lipid accumulation can be toxic and trigger liver injury *via* hepatic parenchymal cell inflammation. These liver functions are mostly measured by serum AST and ALT levels (22). In this study, a significant increase of ALT and AST in serum was observed in the HFD group. Notably, *L. acidophilus* KLDS1.0901 administration conspicuously inhibited this effect. These results demonstrated that *L. acidophilus* KLDS1.0901 administration protects liver functions and mitigates HFD-induced liver injury. Host tissue damage and repair, immune modulation, and inflammation activities are generally regulated by cytokines. Similarly, cytokines such as IL-6, IL-1β, and TNF-α can be mechanistically stimulated by intestinal bacterial communities (23). It has been reported that *Lactobacillus* and *Pediococcus* ameliorate the progression of NAFLD through the modulation of cytokines (24). Our results showed that HFD induction increased the IL-6, IL-1β, and TNF-α concentrations accompanied by the decrease of IL-10. However, *L. acidophilus* KLDS1.0901 administration effectively restored the trend, indicating that *L. acidophilus* KLDS1.0901 administration could alleviate the NAFLD by modulating the concentrations of cytokines.

The gut and the liver communicate through the gut–liver axis, which consists of the gut, the liver, and the intestinal barrier (25). Tight junctions, composed of ZO-1, occludin, and claudin, link the intestinal epithelial cells and maintain intestinal barrier integrity (26). Both intestinal barrier malfunction and dysbiosis of the gut microbiota play important roles in the pathophysiology of liver diseases (27). NAFDL-associated gut microbiota dysbiosis induced by long-term consumption of a high-fat and high-fructose diet may disrupt intestinal barrier function by reducing the expression of intestinal tight junction proteins (occludin, claudin-1, and ZO-1) (28).

Homeostasis in intestinal epithelial cell permeability and regulating barrier functions are modulated by ZO-1 (29), with occludin serving as a tight junction protein molecule (30). Claudins and mucins (Muc1–Muc6), on the contrary, regulate inflammation, intestinal epithelial homeostasis, and the colon mucus layer (31, 32). Intestinal inflammation typically results when the absence of tight junctions weakens the intestinal barriers and allows bacterial invasion (33). Our results showed that the mRNA levels of these four genes were strikingly downregulated in the HFD group, but *L. acidophilus* KLDS1.0901 administration upregulated the mRNA levels of these four genes. Intestinal inflammation results in “leaky gut,” a condition where bacteria and their metabolites trigger the release of cellular inflammatory factors with deleterious effects on the host (34). Several previous investigations have demonstrated that LPS regulates intestinal flora and inflammation responses involved in the onset of metabolic diseases (35). In particular, NAFLD onset has been correlated with the presence of bacterial LPS from enteric gram-negative flora (53). In this study, the concentration of LPS and D-lactic acid was significantly increased (p < 0.05) in the HFD group, but *L. acidophilus* KLDS1.0901 administration could drastically reduce the concentration of LPS and D-lactic acid. These findings indicated that *L. acidophilus* KLDS1.0901 administration could improve the intestinal barrier function induced by HFD.

The gut microbiome is a key environmental factor in the onset of NAFLD (36). Firmicutes and Bacteroidetes are essential participants in host energy metabolism (37). The phylum-level study of a mixed lactobacilli treatment administered to HFD-fed mice indicated that the Firmicutes bacterial group increased with a corresponding drop in the relative abundance of Bacteriodetes (38–40). These researchers discovered that an increased Firmicutes to Bacteriodetes ratio increased calories absorption, harvestable energy levels, and obesity biomarkers (41, 42), which was in line with the results of Yu et al. (15). The results showed that the F/B ratio was significantly lower in the *L. acidophilus* KLDS1.0901 group, suggesting that 1.0901 may regulate energy metabolism in mice by modulating intestinal flora, which, in turn, affects the degree of fat accumulation.

The genus-level gut microbiota results for the different groups are shown in Figure 8B. We observed that in the HFD group, the relative abundances of *Roseburia, Lachnospiraceae UCG-006, Bacteroides*, and *Enterorhabdus* were much higher when compared to the NC group, which were suppressed by *L. acidophilus* KLDS1.0901 administration. However, the HFD group had low levels of *Blautia, Alistipes, Oscillibacter, Faecalibaculum, Ruminiclostridium, Lactobacillus*, and *Ruminococcaceae UCG-009*, which were reversed by *L. acidophilus* KLDS1.0901 administration. Our observations indicated that *L. acidophilus* KLDS1.0901 administration modulated the composition of the intestinal microbiome of HFD-fed mice.

Genus-level results of the current study indicate that the relative abundances of *Roseburia, Lachnospiraceae UCG-006, Bacteroides*, and *Enterorhabdus* elevated while that of *Blautia, Alistipes, Oscillibacter, Faecalibaculum, Ruminiclostridium, Lactobacillus*, and *Ruminococcaceae UCG-009* were significantly decreased in the HFD group when compared to the NC group. It has been reported that *Roseburia*, which is known for suppressing SCFAs-producing bacteria, was enriched in HFD-induced NAFLD mice (43, 44). *Enterorhabdus* secretes LPS and is thus implicated in obesity, insulin resistance, and pro-inflammatory factors proliferation (21). Moreover, a substantial relative abundance of *Bacteroides* has been observed in individuals with NAFLD (45). Conversely, SCFAs secreted by, *Blautia*, is known to be pathogen-inhibiting and promotes healthy intestinal microbiota (46). *Alistipes* was negatively correlated with obesity (47). *Lactobacillus* is known to be pathogen-inhibiting and promotes healthy intestinal microbiota (48). Previous observations note that HFD reduced the relative abundance of *Faecalibaculum* (49). In addition, *Ruminiclostridium* were linked to weight reduction or a lean phenotype (50). In summary, *L. acidophilus* KLDS1.0901 further alleviates NAFLD through the intestinal-liver axis by regulating the ecological imbalance of intestinal microbiota caused by a high-fat diet and improving intestinal barrier function.

## Conclusion

*Lactobacillus* KLDS1.0901 administration could significantly decrease body weight gain, Lee's index body, fat rate, and liver index. *Lactobacillus* KLDS1.0901 administration could significantly improve lipid profiles by decreasing the hepatic levels of TC, TG, and LDL-C and ALT and AST in serum and increasing the HDL-C levels. *L. acidophilus* KLDS1.0901 administration could decrease the IL-6, IL-1β, and TNF-α concentrations accompanied by the decrease of IL-10. *L. acidophilus* KLDS1.0901 administration could improve the intestinal barrier function by upregulating the mRNA levels of occludin, claudin-1, ZO-1, and Muc-2, which were coupled to the decreases of the concentration of LPS and D-lactic acid. Notably, *L. acidophilus* KLDS1.0901 administration modulated the gut microbiota to the normal pattern. Hence, our study provides guidance for the selection and application of presumed probiotics in the treatment of NAFLD.

## Data availability statement

The data presented in the study are deposited in the NCBI repository (https://www.ncbi.nlm.nih.gov/sra/), accession number PRJNA925467.

## Ethics statement

The animal study was reviewed and approved by Northeast Agricultural University Animal Ethics Committee.

## Author contributions

BL and BT designed the study. YWan and FJ performed the experiments. YWang and ZW wrote the manuscript. XS and ZX analyzed the data. All authors contributed to the article and approved the submitted version.
