# Supplementary material for: Assessing the in vivo ameliorative effects of Lactobacillus acidophilus KLDS1.0901 for induced non-alcoholic fatty liver disease treatment
Source: Front Nutr. 2023 Mar 20;10:1147423. doi: 10.3389/fnut.2023.1147423 (PMC10067668; doi:10.3389/fnut.2023.1147423)
Supplement: Supplementary file 1 [file Data_Sheet_1.docx]

**Table S1** Feed formula of mice

| Ingredient | D12450B control feed (g/kg) | D12492 high fat feed (g/kg) |
| --- | --- | --- |
| Casein, 80mesh | 200 | 200 |
| L-Cystine | 3 | 3 |
| Corn starch | 315 | 0 |
| Maltodextrin 10 | 35 | 125 |
| Sucrose | 350 | 68.8 |
| Cellulose BW20 | 50 | 50 |
| Soybean oil | 25 | 25 |
| Lard | 20 | 245 |
| Mineral mix S10026 | 10 | 10 |
| Dicalcium phosphate | 13 | 13 |
| Calcium carbonate | 5.5 | 5.5 |
| Potassium citrate, 1 H_2_O | 16.5 | 16.5 |
| Vitamin mix V10001 | 10 | 10 |
| Choline bitartrate | 2 | 2 |
| FD&C yellow dye # 5 | 0.05 |  |
| FD&C blue dye # 1 |  | 0.05 |

**Table S2** NAFLD activity integral

| Item | Score | Definition |
| --- | --- | --- |
| Steatosis | 0 | <5% |
|  | 1 | 5-33% |
|  | 2 | >33%-66% |
|  | 3 | >66% |
| Hepatocyte  Ballooning | 0 | None |
|  | 1 | Few balloon cells |
|  | 2 | Many cells/prominent ballooning |
| Lobular Infla  mmation | 0 | No foci |
|  | 1 | <2 foci/x200 field |
|  | 2 | 2-4 foci/x200 field |
|  | 3 | >4 foci/x200 field |

**Table S3** Forward and reverse primer sequences for quantitative real-time PCR.

| Genes | Forward (5′-3′) | Reverse (5′-3′) |
| --- | --- | --- |
| GAPDH  Claudin1 | GGTTGTCTCCTGCGACTTCA  GCTGGGTTTCATCCTGGCTTCTC | TGGTCCAGGGTTTCTTACTCC  CCTGAGCGGTCACGATGTTGTC |
| Occludin | TTGGCTACGGAGGTGGCTATGG | CCTTTGGCTGCTCTTGGGTCTG |
| ZO-1 | AACCCGAAACTGATGCTGTGGATAG | CGCCCTTGGAATGTATGTGGAGAG |
| Muc-2 | TGCTGACGAGTGGTTGGTGAATG | TGATGAGGTGGCAGACAGGAGAC |
